# A 3D Printed Toolbox for Opto-Mechanical Components

**DOI:** 10.1371/journal.pone.0169832

**Published:** 2017-01-18

**Authors:** Luis José Salazar-Serrano, Juan P. Torres, Alejandra Valencia

**Affiliations:** 1 Quantum Optics Laboratory, Universidad de los Andes, Bogotá, Colombia; 2 ICFO - Institut de Ciencies Fotoniques, The Barcelona Institute of Science and Technology, Castelldefels (Barcelona), Spain; 3 Dep. of Signal Theory and Communications, Universitat Politecnica de Catalunya, Barcelona, Spain; Universidad Miguel Hernandez de Elche, SPAIN

## Abstract

In this article we present the development of a set of opto-mechanical components (a kinematic mount, a translation stage and an integrating sphere) that can be easily built using a 3D printer based on Fused Filament Fabrication (FFF) and parts that can be found in any hardware store. Here we provide a brief description of the 3D models used and some details on the fabrication process. Moreover, with the help of three simple experimental setups, we evaluate the performance of the opto-mechanical components developed by doing a quantitative comparison with its commercial counterparts. Our results indicate that the components fabricated are highly customizable, low-cost, require a short time to be fabricated and surprisingly, offer a performance that compares favorably with respect to low-end commercial alternatives.

## Introduction

Nowadays is not uncommon to find headlines in the media where it is stated that 3D printing is a technology called to change our lives in the near future [1, 2]. For many authors, we are living the time of a third industrial revolution [3, 4]. However, we are currently in a stage of development where the use of 3D printing is advantageous over other manufacturing technologies only in rare scenarios. Fortunately, scientific research is one of them [5].

The implementation of opto-mechanical components using 3D printing technology is having a direct impact on the photonics community [6, 7]. Researchers are no longer constrained to work with commercially available products and therefore their experimental setups can be made more versatile. Thanks to the fact that the fabrication time is minimal, the characteristics and quality of the components can evolve very fast in the hands of researchers. Indeed this evolution process turns out to be a creative way to engage young researchers in photonics. Moreover, the new 3D printed components can be considered as prototypes, and thus other manufacturers can take these ideas to build better equipment or improve their own products.

A nice example to illustrate this is the Open-Source Optics Library [8]. A library of opto-mechanical components built from standard low-cost parts and 3D printed components aimed at providing a flexible and cost-effective alternative to current commercial alternatives. The library is composed of a broad selection of optical components ubiquitous in any optics experiment such as a lens holder [9], a screen/filter holder [10, 11], a lab jack [12], a fiber optic holder [13], a kinematic mirror/lens mount [14], a parametric open-source chopper wheel [15], among others. To arrange the components in a particular configuration, the standard optical table is exchanged by a 8mm smooth rod from which each element is fixed [16–19] or by a set of magnetic bases [20] that can be fixed to any already available steel plate. Since each component 3D printed is a parametric design in OpenSCAD [21], it can be easily modified, improved or customized by changing a few lines of code.

Interestingly, more advanced equipment is also accessible by combining 3D printed components with an Arduino or a Raspberry pi card that provides sensing, processing and actuation capabilities. This is the case of the open-source colorimeter [22] used for determining the concentration of dissolved species, the DIYbio centrifuge [23] used for DNA extraction, the open-source syringe pump [24] developed to provide a carefully controlled dose of a given reagent, the open-source quartz crystal microbalance [25] capable of measuring phenomena at molecular scale in fluids, vacuum or air, or the Smartphone laser beam spatial profiler [26] that can be used to characterize easily the spatial profile of a laser beam, just to name a few. In all these cases, the gap of overly engineered components for experimental setups is filled with an equipment that despite costing a fraction of its commercial counterpart can be used to perform state-of-the-art research.

In this paper we report the design and construction of a set of opto-mechanical components intended to complement the library of open-source optical components already available on the web [8, 27, 28]. In our approach, we put special emphasis on evaluating the performance of each component by carrying out a quantitative comparison with respect to its commercial counterpart. The components are developed with 3D printers based on FFF [29] that can be used in various experimental setups in photonics, either for research or teaching. In particular, we report results for a kinematic mount, a translation stage, and an integrating sphere.

Since 3D printers based on FFF are becoming more affordable, the opto-mechanical components of our set can be fabricated practically in any location. It is important to highlight that since the components fabricated have a similar performance than low-end commercial alternatives in terms of stability and robustness, the approach presented here makes photonics more accessible to industry and academia in regions and countries with scarce resources, enlarging significantly the size of the community interested in performing experiments in photonics and related fields [30]. The ideas presented here also constitutes a new path to reduce increasingly rising costs, and in certain places can constitute a way to overcome funding restrictions and large lead times due to customs and administrative procedures [6].

## Materials and Methods

We have designed and fabricated a set of opto-mechanical components that are ubiquitous in any optics laboratory either for research or teaching. For the sake of illustration we have fabricated all the opto-mechanical components necessary to construct a Michelson interferometer, since it is an experimental setup that requires a very diverse set of opto-mechanical components for its implementation.

**Fig 1:**
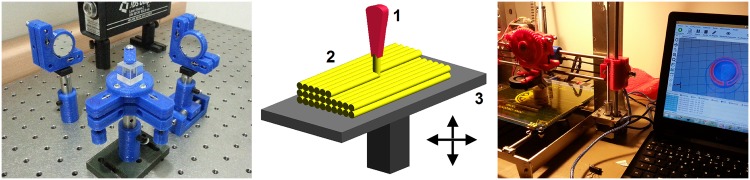
Set of printed opto-mechanical components. (A) A Michelson interferometer implemented with opto-mechanical components made in plastic. (B) Fused Filament Fabrication (FFF), a method of rapid prototyping: 1. Nozzle ejecting molten material (plastic), 2. Deposited material (modelled part), 3. Controlled movable table. (C) Printing a component using a Prusa-Tairona printer.

In Fig 1A we depict some of the components fabricated, such as **kinematic mounts**, used to set the tip and tilt of mirrors and lenses, **translation stages**, used to set the position of a component along a single axis with high precision, **kinematic platforms**, used to support and set the position of components such as prisms or beam splitters. Moreover, we have also built an **integrating sphere**, used to measure the power of a light source, and other basic components such as **post holders** and **post clamps**. All the plastic parts have been printed using a Prusa-Tairona [31] 3D printer that cost ∼400€ and was manufactured in Colombia.

The fabrication process used to build the opto-mechanical components consists of two parts. In the first part, the main elements are built in plastic using a standard 3D fabrication process, and in the second part the construction of the opto-mechanical components is implemented.

The first part can be described as follows. To begin with, a 3D model of the component is designed using a CAD software, such as OpenSCAD, Blender or Solid Works. If available, the design can be downloaded from a digital design repository like Thingiverse [32]. For the opto-mechanical components reported here, all the plastic components were designed using OpenSCAD [21], an open-source, script-based, user-friendly software that generates 3D models by combining (adding or subtracting) primitive shapes such as cylinders, spheres and cubes. After the design is finished, it is converted, using another software like slicer or cura, into printing instructions for the 3D printer. In our case, the Prusa Tayrona printer uses the programs Repetier Host [33] and Slicer to generate the gcode and print the piece, respectively.

The components are fabricated using an additive manufacturing technology known as Fused Filament Fabrication in which a filament of PLA (Polylactic Acid, a biodegradable thermoplastic polymer made from plant-based resources such as corn starch or sugar cane) is heated and then extruded through a hot nozzle. The hot plastic is deposited layer by layer following a given pattern so that each layer binds with the layer below to build a solid object. A moving platform or moving nozzle determines the position of the hot filament and thus the shape of the solid object printed (see Fig 1B and 1C).

**Fig 2:**
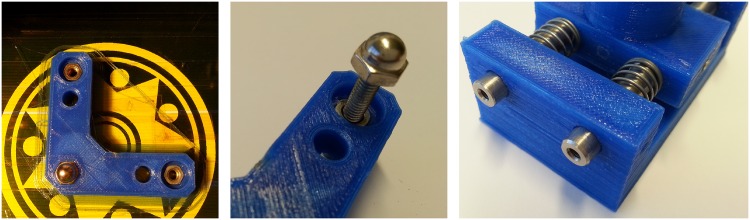
Mechanism Implementation. (A) Nuts of different shapes embedded on the plastic immediately after fabrication. (B) Drive screw mechanism implemented using a nut embedded in the plastic and a screw. The precision in the displacement is limited by the nut’s thread. (C) Linear bearings can be replaced by holes carefully made in plastic through which passes a metallic rod.

Once the main components are printed, the second part of the construction process begins. The plastic elements are combined with components like nuts, screws, bolts, washers, springs and rods, easily found on a hardware store, to create a fully functional opto-mechanical component. Fig 2A and 2B depict a drive screw mechanism implemented by embedding a nut in the plastic. Fig 2C shows the implementation of a linear bearing using a rod. It is remarkable that even though the components added in this second part are not specifically designed for high precision applications, we found that the opto-mechanical components fabricated with them provide a very similar performance when compared with its commercial counterparts, as it is shown in the Results section.

### Kinematic Mount / Kinematic Platform

**Fig 3:**
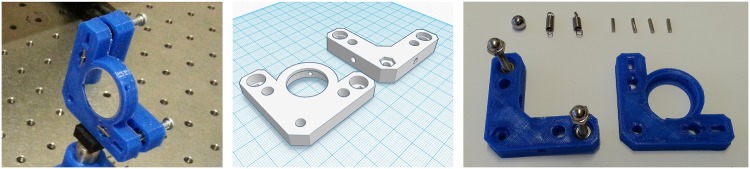
3D printed kinematic mount. (A) Finished kinematic mount with mirror mounted. (B) 3D model of plastic components (KM top: leftmost component, KM bottom: rightmost component). (C) Components required to build the kinematic mount.

A kinematic mount (KM) is an opto-mechanical component used in optical laboratories to adjust with high precision the tip and tilt of a mirror (or lens), while it holds the component securely in place, as shown in Fig 3A. A kinematic platform (KP) may be seen as a rotated kinematic mirror mount that is mainly used to control the tip and tilt of a flat surface where other components such as prisms, beam splitters or non-standard optics are secured.

Our proposed implementation of the kinematic mirror mount, follows the widely used cone, groove, and flat constraint scheme and is based on the original design of Doug Marett [34] that can be found on Thingiverse. The mount is implemented by printing two pieces of plastic (Fig 3B) that are joint together using a sphere and two springs secured with two rods on each side. The drive screw mechanism is built using two nuts that are embedded into the plastic. Two M4 screws with rounded nuts in one end are used to adjust the tip and tilt respectively (Fig 3C). The rounded nuts are used to keep the two plastic pieces in position and to reduce any unwanted motion. A complete list of the required materials is shown in Table 1.

**Table 1:** Bill of Materials for Kinematic Mount.

Component	Comments	Quantity	Unit cost [€]
KM top	Vol 9.663 cm^3^	1	4.8
KM bottom	Vol 10.588 cm^3^	1	5.3
Steel sphere	*ϕ* = 8 mm	1	0.25
Hex nut	M4	4	0.25
Rounded nut	M4	2	0.25
Screw	M4, *L* = 4 cm	2	0.25
Tension spring	*L* = 1 cm	2	0.25
Metal rod	*L* ≈ 1 cm	2	0.25
		**TOTAL**	**13.35**

Typically, a mirror mount may cost between 35€, for a basic mount, to 150€, for a more advanced component. These values correspond to average costs calculated from available data of some popular manufacturers such as Newport, Thorlabs and Edmund Optics. Moreover, the lead time may vary between 2 and 7 days for locations in Europe or United States, to between 1 and 4 months for locations in other countries (for instance, 4 months may be typical for an university lab in Colombia). It is clear that with plastic as fundamental material we cannot outperform other high-performance components, mainly due to the limitations imposed by the plastic mechanical properties. However, we can still reproduce the behaviour of low-end opto-mechanical components at a fraction of the cost and with a significant reduction in lead time to a few hours.

The kinematic mount shown in Fig 3A was built in three hours: the first two hours were spent printing the two plastic components, and the last hour was spent building, adjusting and testing the component. The total cost of manufacture is 12€ (a printing cost of 0.5€/cm^3^ is assumed), including the components purchased in a hardware store. As a result the cost is significantly reduced, but more importantly, the lead time is dramatically reduced from days or months to hours. KM top and KM bottom component files are available on Thingiverse [35].

### Translation stage

**Fig 4:**
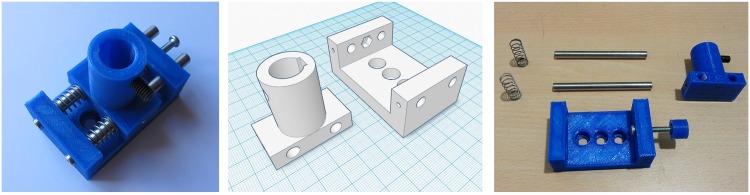
3D printed translation stage. (A) Finished translation stage. (B) 3D model of plastic components (TS top: leftmost component, TS bottom: rightmost component). (C) Components required to build the translation stage.

A translation stage (TS) is a component typically used to vary the position of an object along a single axis. The position of the moving platform is controlled with a drive screw mechanism more precise that the one used in the kinematic mount (see Fig 4A).

The translation stage is composed of the two pieces shown in Fig 4B. Unlike a traditional system, where the rods are fixed and the moving section has two linear bearings, the stage printed has the two rods fixed to the moving section, and the walls of the main platform act as bearings. With this scheme it was possible to obtain a very stable and robust linear motion along the axis of the rods. The drive screw mechanism is built using a single nut that is embedded in one wall of the main platform. A M4 screw with a rounded nut in one end is used to set the position of the moving section over a range of 1.0 cm.

According to our experience, a standard translation stage cannot be found for less than 150€. This amount can represent a considerable investment for a laboratory or company that is on an early stage of development. Regarding the lead time, there is no much difference with respect to the kinematic mount presented above.

The component shown in Fig 4A was built in four hours. Three hours to print and clean the plastic components and one hour to leave the stage fully operational. In Table 2 there is a list of the required materials and the corresponding manufacturing costs. TS top and TS bottom component files are available on Thingiverse [36]. A printing cost of 0.5€/cm^3^ is assumed.

**Table 2:** Bill of Materials for Translation Stage.

Component	Comments	Quantity	Unit cost [€]
TS top	Vol 14.848 cm^3^	1	7.4
TS bottom	Vol 9.663 cm^3^	1	16.3
Steel rod	*ϕ* = 4 mm, *L* ≈ 7.5 cm	2	4
Hex nut	M4	1	0.25
Rounded nut	M4	1	0.25
Screw	M4, *L* = 4 cm	1	0.25
Grub screw	M6, *L* = 1 cm	1	0.25
Spring	*L* = 1.5 cm	2	0.25
		**TOTAL**	**33.2**

### Integrating sphere

An integrating sphere is an optical component composed of a hollow spherical cavity that has two small windows orientated at 90° with respect to each other. The first window corresponds to the input port whereas the other is the output port where a detector is located. For commercial devices, the interior of the sphere is covered with a diffuse reflective coating. When light enters into the sphere, it is reflected equally in all directions due to scattering. As a result, an integrating sphere is a device that can be used to measure optical power, while the spatial information of the input beam is erased, i.e., beam shape and entrance angle.

**Fig 5:**
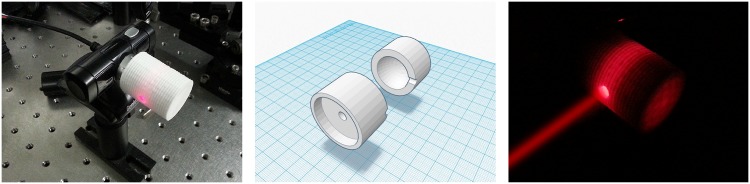
3D printed integrating sphere. Panel (A). Integrating sphere using a webcam as a detector. Panel (B). 3D model of the plastic integrating sphere. The inner sphere is printed as a complete piece. In the figure the sphere is divided in two sections for illustrative purposes. Panel (C). Integrating sphere operating when the laboratory lights are turned off.

Fig 5A shows the 3D printed integrating sphere connected to a webcam. Fig 5B shows a model of the internal structure of the device. Fig 5C shows the sphere in operation when a He-Ne beam is aimed at the input port, and the laboratory lights are turned off. In our design the output window allows to use any of the following detectors: photodiode, webcam, or the camera of a mobile phone.

In order to calibrate and test the performance of our integrating sphere using a webcam as a detector, we compare its output power reading with respect to the one of a commercial power meter (Thorlabs S130C connected to a PM100 Analog Handheld Laser Power Meter Console). This was done by using a program written in Python that calibrates the device with respect to a known reference and also can be used to perform power measurements.

In order to determine the light input power from the webcam, each image taken is first converted to a matrix and then filtered using a circular mask of a given radius *R*. Subsequently an intensity histogram is generated using the function calcHist available in OpenCV [37]. The area below this histogram is proportional to the input power.

The component shown in Fig 5A was built in two hours, where all the time was spent printing the components. Table 3 shows the elements required and the total cost of implementation. Notice that the final cost corresponds to an overwhelming reduction of more than 95% with respect to the commercial equipment used to characterize the device which cost ∼1300€. However, as discussed below, it has to be taken into consideration that the range of operation of the 3D printed detector is very limited since the maximum power it can measure, before saturation of the camera occurs, is 0.3 mW. As a result, the device described is suitable for undergraduate laboratories. IS top and IS bottom component files are available on Thingiverse [38]. A printing cost of 0.5€/cm^3^ is assumed.

**Table 3:** Bill of Materials for Integrating Sphere.

Component	Comments	Quantity	Unit cost [€]
IS top	Vol 20.475 cm^3^	1	10.2
IS coupler for webcam	Vol 1.6 cm^3^	1	0.8
Webcam	model Genius eye 110	1	20
		**TOTAL**	**31**

## Results

**Fig 6:**
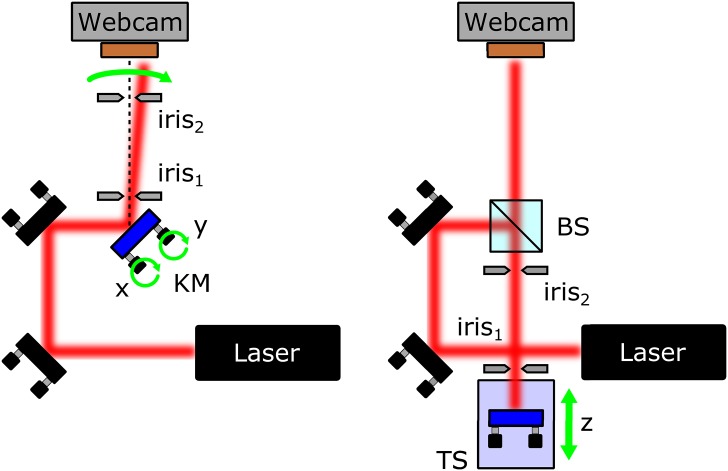
Experimental setup used to characterize 3D printed components. (A) Setup used to compare two kinematic mounts; KM kinematic mount under test. (B) Setup used to compare two translation stages; TS translation stage under test.

To determine the performance of the opto-mechanical components fabricated, a direct comparison with respect to its commercial counterparts was carried out by using the experimental setups presented in Fig 6.

Fig 6A shows the scheme used to measure the performance of the kinematic mount. The input beam is generated using a He-Ne laser with a Gaussian spatial beam profile with beam waist of ∼1 mm. After two reflections, the beam is reflected in a mirror mounted on the kinematic mount to be tested (indicated in blue) and its centroid position is monitored using a webcam located at 80 cm along the mirror vertical axis. A routine written in Python, that uses the OpenCV library, records the beam centroid as a function of the angle of the kinematic mount. The beam reference position is determined by aligning initially the beam with respect to the two irises 1 and 2 located before the camera.

To test the performance of the translation stage, the setup shown in Fig 6B is used. The beam reflected by the beam splitter (BS) is reflected by a mirror located in the translation stage to be characterized. The TS is positioned in such a way that the beam reflected passes through the two irises for different positions.

**Fig 7:**
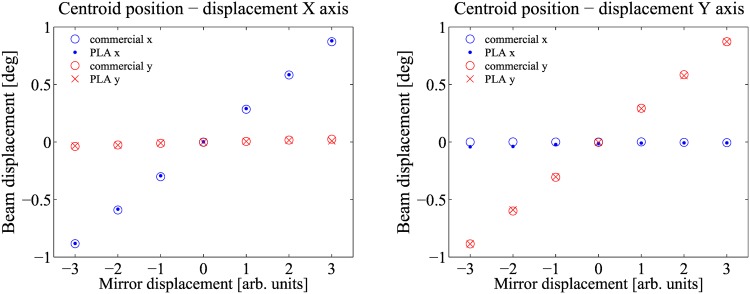
Experimental results kinematic mount. Centroid position as a function of the X knob (A) and Y knob (B).

In order to evaluate the performance of each opto-mechanical component, two sets of measurements were carried out. The first set is taken using the 3D printed component and the second one using a commercial device. For the kinematic mount, the interval (−1°, +1°) in the horizontal and vertical directions is divided equally in seven points. Each direction is scanned ten times by rotating either the X or Y knob, in order to evaluate the hysteresis and repeatability of the component. Fig 7A and 7B show the centroid position as a function of the X or Y knob rotation, respectively. In both cases, it is clearly seen that the 3D printed kinematic mount, exhibits the same behaviour as its commercial counterpart, in this case a Thorlabs KM100 mount. In fact, when the beam is shifted in one direction, the other is confined within the same small interval as the commercial component. From the characterization, the only difference observed between both components appears in the sensitivity experienced in the knob rotation, determined by the screw thread.

**Fig 8:**
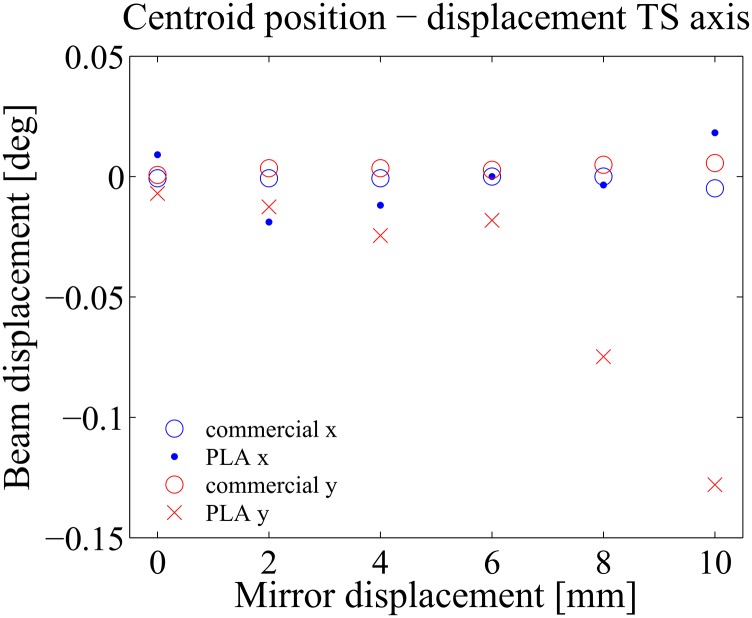
Experimental results translation stage. Centroid position as a function of the mirror displacement in millimeters.

For the translation stage, two sets of measurements where performed over the interval (0 mm, +10 mm) with the translation step set at 2 mm. The results are shown in Fig 8. From the results is observed that for the commercial TS (Standa 7T173-20-50), the beam drift lies within the interval (−0.01°, +0.01°). On the other hand, the 3D printed TS performs worse, particularly for displacements beyond 6 mm, where the rounded screw exerts a significant pressure on the moving platform. This pressure gives rise to the unwanted displacement observed in the y-direction. Fortunately, for small translations, below 6 mm, the device presents an acceptable performance where the beam experiences a drift that lies within an interval of tenths of degrees. Notice that this unwanted beam displacement is imperceptible to the eye.

**Fig 9:**
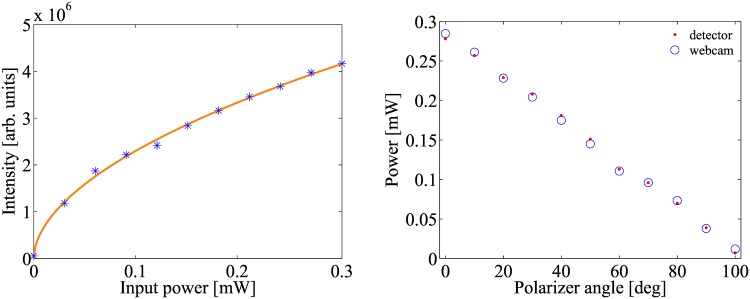
Experimental results integrating sphere. (A) device response as a function of the intensity (area behind image histogram) for *R* = 100*pixels*. (B) Comparison with a commercial power meter after calibration. Maximum error 0.6*μ*W.

Regarding the integrating sphere, it is found that it exhibits a non-linear response as a function of the input beam intensity. Fig 9A displays an example of the device response as a function of the input intensity. From the fitted data, a calibration curve is obtained that is further used to determine the response of the power meter. For the sake of example, Fig 9B presents a single wavelength (λ = 633*nm*) comparison between a commercial power meter and our 3D printed integrating sphere using a webcam as a detector. From the data we can deduce a maximum relative error of less than 2%. In the experiment the light intensity is controlled by changing the angle between two crossed polarizers.

## Discussion

3D printing is a technology that is called to change our lives in the near future, by changing paradigms in the way that many things are fabricated nowadays. It offers the possibility to lower significantly the cost of production and to reduce dramatically delivery times. An important consequence of this is the opportunity that opens to countries, companies and individuals with less economic power to enter new fields of technology, such it is the case of Photonics, not only for research but also for building and testing new prototypes with potential market value.

In this work we report the design, fabrication and characterization of a set of opto-mechanical components that are essential in any optics laboratory either for research or teaching. Not surprisingly, the methodology used provides a way to reduce dramatically the cost (between 50% and 90% depending on the component) of a standard kinematic mount, a basic translation stage and an integrating sphere with respect to commercial components. However, even though the components fabricated were not primarily designed for high precision applications, we have found that they provide very similar performance with respect to its low-end commercial counterparts.

As a result, the components presented here are suitable for experimental setups where transverse beam stability, directly related to the rigidity of the material used to implement the opto-mechanical component, is not a crucial factor and thus PLA can be used. Notice that other materials such as ABS or nylon may be used to fabricate the 3D components since special attention has to be taken if PLA is used since its mechanical properties may change in a humid environment.

From the characterization we observe no relevant difference for the kinematic mount in terms of hysteresis and repeatability when compared with a Thorlabs KM100 kinematic mirror mount. In addition, for the translation stage, we identify a regime of operation where the beam drift lies within commercial limits defined by the Standa 7T173-20-50 linear translation stage. We believe that this regime can be improved by introducing plastic linear bearings [39] in the moving platform, or by optimizing its geometry (width and height). Lastly, regarding the integrating sphere we found that it provides a linear response for input powers below 0.3 mW. We estimate that this value can be further increased to reach hundreds of milliwatts by optimizing the geometry of the component in terms of inner diameter, input and output window diameters and wall thickness.

## Conclusion

We have developed and characterized a set of opto-mechanical components that can be easily implemented using a 3D printer based on Fused Filament Fabrication and mechanical parts that can be found on any hardware store. In particular, we have built three of the main components required to implement a Michelson interferometer, namely a kinematic mount, a translation stage and an integrating sphere. We have also compared its performance against some selected available commercial alternatives.

Our results indicate that 3D printing provides a suitable alternative to implement experimental equipment in scenarios where is not required extremely high precision. Somehow surprisingly, our 3D printed kinematic mount provides a very similar performance with respect to its commercial counterpart. Even though the results obtained for the translation present an imperceptible to the eye beam drift, the device can be suitable for experiments in undergraduate laboratories. Regarding the integrating sphere, we have developed and demonstrated a simple accessory that can be printed in order to convert a webcam into a power detector.

Importantly, in all cases we have found that a 3D printer is an extremely useful resource in any laboratory since it opens the possibility to fabricate experimental equipment that is highly customizable, at a low cost with respect to commercial alternatives, and more importantly in a very small period of time.

## Supporting Information

S1 File Experimental data from the characterization of 3D printed opto-mechanical components. Data corresponding to the measurements presented in the Results section where the kinematic mount, translation stage and integrating sphere are characterized.
(ZIP)
